# Factors Associated With Dyskinesia in Parkinson's Disease in Mainland China

**DOI:** 10.3389/fneur.2019.00477

**Published:** 2019-05-15

**Authors:** Xun Zhou, Jifeng Guo, Qiying Sun, Qian Xu, Hongxu Pan, Renhe Yu, Jieqiong Tan, Xinxiang Yan, Beisha Tang, Liangjuan Fang

**Affiliations:** ^1^Department of Neurology, Xiangya Hospital, Central South University, Changsha, China; ^2^Laboratory of Medical Genetics, Central South University, Changsha, China; ^3^National Clinical Research Center for Geriatric Disorders, Changsha, China; ^4^Key Laboratory of Hunan Province in Neurodegenerative Disorders, Central South University, Changsha, China; ^5^Center for Brain Disorders Research, Beijing Institute for Brain Disorders, Capital Medical University, Beijing, China; ^6^Department of Geriatric Neurology, Xiangya Hospital, Central South University, Changsha, China; ^7^School of Public Health, Central South University, Changsha, China

**Keywords:** Parkinson's disease, levodopa, dyskinesia, risk factors, disease duration

## Abstract

**Background and Objectives:** Studies examining the risk factors for dyskinesia in Parkinson's disease (PD) have been inconsistent, and racial differences exist. Since there have been no systematic studies of the characteristics of dyskinesia in the Mainland Chinese population, we sought to elucidate the risk factors for dyskinesia.

**Methods:** A total of 1974 *PD* patients from Mainland China were systematically investigated by univariable and multivariable analyses. PD patients with and without dyskinesia were stratified into 4 groups according to levodopa equivalent daily dose (LEDD) and analyzed by a Cox proportional hazards model. A longitudinal study of 87 patients with dyskinesia was classified into 3 groups according to the duration from onset of PD to the initiation of levodopa, and comparisons among groups were analyzed by the Mann-Whitney test.

**Results:** Early age of onset, long disease duration, being female, high LEDD, low UPDRS III scores (ON-state) and high Hoehn-Yahr stage (ON-state) were predictors of dyskinesia. Dyskinesia was levodopa dosage-dependent, and the incidence increased remarkably when LEDD exceeded 300 mg/d (*p* < 0.05). The emergence of dyskinesia had no association with the initiation time of levodopa, and if the latter was more than 4 years, the duration of time on chronic levodopa free of motor complications was significantly shortened.

**Conclusions:** We found risk factors for the prediction of dyskinesia. Our data shows that physicians should be cautious if the LEDD exceeds 300 mg/d. The development of dyskinesia was not correlated with the time of levodopa initiation.

## Introduction

Parkinson's disease (PD) is the second most common neurodegenerative disorder and is characterized by inexorably progressive depletion of dopaminergic neurons in the substantia nigra (SN) and subsequent loss of dopamine in the dorsal striatum, leading to classical motor deficits, including bradykinesia, tremor, rigidity, and postural instability. Levodopa, as a compensation for dopamine loss, remains the gold standard of treatment. However, with disease progression and longer exposure to levodopa, patients develop a range of motor complications, including dyskinesia that negatively impact quality of life and impose a significant economic burden. Dyskinesia develops progressively, and the rates observed in clinical trials range from 33 to 54% after 4–6 years of levodopa treatment (1, 2) and from 52 to 71% after 10 years (3, 4). However, the first multicenter epidemiological data of dyskinesia among PD patients on levodopa therapy from Mainland China indicated that the overall dyskinesia prevalence rate was 10.3% (5). Although some benefits have been shown with amantadine ER, continuous dopaminergic stimulation and deep brain stimulation (DBS), there are significant side effects and restrictions (6, 7). Therefore, delaying or preventing the occurrence of dyskinesia is particularly important.

Several factors have been associated with the development of dyskinesia, including duration of levodopa exposure, LEDD, initial levodopa dosage, disease severity, and age of onset (8, 9). Although the ELLDOPA study did not show any evidence that early use of levodopa negatively correlated with dyskinesia, the pattern of treatment was not changed due to concern for motor complications on the part of neurologists (10). In addition, a few clinical trials reported that initial treatment with dopamine receptor agonists could reduce dyskinesia (1). Other risk factors, such as genetic predisposition, sex, and body mass index (BMI) might participate in the development of dyskinesia (11–13). The results from published studies have been inconsistent, and racial differences exist. Recognition of the full characteristics of dyskinesia is essential for adequate diagnosis and management.

Over the past decades, there have been no systematic studies to characterize dyskinesia in the Chinese population. In the current study, we collected data from a large sample of PD patients from Mainland China and investigated the association between dyskinesia and related risk factors, especially the medication dosage. We also performed a longitudinal study to explore whether the emergence of dyskinesia was correlated with the initiation time of levodopa.

## Methods

### Study Design and Participants

All subjects (*N* = 1,974) were consecutively included during the period between February 2017 and June 2018 in different regions of Mainland China. Each patient was diagnosed with clinically established PD or clinically probable PD by at least two experienced neurologists according to movement disorder society (MDS) diagnostic criteria (14). In cases of any doubt or uncertainty, the patient was admitted to the hospital for an in-depth clinical evaluation and additional investigation (e.g., neuroimaging, electromyography, etc.). Key exclusion criteria included other Parkinsonism, significant neurological or psychiatric disorder, previous surgery or DBS for PD, no levodopa treatment history or treatment discontinuation. The presence of dyskinesia, including peak-dose dyskinesia, OFF-state dystonia and diphasic dyskinesia based on either history or direct observation, was assigned to the dyskinesia group (*n* = 336) and was compared with patients without dyskinesia (*n* = 1,638).

Anti-parkinsonism medication histories were recorded in detail, including types and doses at assessment and at important time points, including onset of wearing-off and dyskinesia. The LEDD was calculated according to Tomlinson et al. (15). Of the 1974 PD patients, 336 patients experienced dyskinesia. Dyskinesia patients who were unaware of the accurate emergence time of dyskinesia (*n* = 21) or who were without clear medication history (*n* = 16) were excluded. To evaluate the effect of LEDD on the risk of developing dyskinesia, 1,937 participants (37 patients were excluded) were stratified into four groups based on the actual LEDD at the onset of dyskinesia or at the evaluation if the patient had not experienced dyskinesia: Group 1: ≤ 300 mg/day (637, 32.9%); Group 2: 301–600 mg/day (871, 45.0%); Group 3: 601–900 mg/day (316, 16.3%); Group 4: >900 mg/day (113, 5.8%).

To determine the disease duration of PD before medication on the risk of developing dyskinesia, 328 PD patients diagnosed according to UK Brain Bank criteria in the out-patient clinic of the Neurology Department in Xiangya Hospital from 2006 to 2013 were recruited. At baseline, patients who experienced motor complications or refused to follow up regularly were excluded. Basic information was registered, and all patients were followed up and evaluated regularly at least every other year. An extra follow-up visit was scheduled if the participant had any signs of motor complications. Motor complication were recorded by clinical observation or assessed by UPDRS IV. The initiation time of wearing-off or/and dyskinesia were recorded. Finally, 87 patients developed dyskinesia during 5–12 years of follow-up with at least ≥1 year intervals. Patients with dyskinesia were classified into 3 groups according to the time from onset of PD to the initiation of levodopa: group A: < 2 years; group B: 2–3 years; group C: ≥4 years.

### Clinical Assessment

Onset of PD or dyskinesia was defined as the year in which a cardinal sign was first noted by the patients, family members or investigators. The duration of PD or dyskinesia was defined as the period between the onset and the time of evaluation. Systematic data including age, sex, disease duration, family history, education level, exposure to pesticides, and personal preferences (cigarette, tea, coffee, wine) were obtained by interview. All patients subsequently underwent extensive neurological assessments performed by experienced investigators in movement disorders who were specifically trained before the evaluation. The assessments included the following: (1) Motor symptoms: Unified Parkinson's Disease Rating Scale (UPDRS) part II and part III; Subtype according to UPDRS: tremor dominant, postural instability and gait difficulty, intermittent; Hoehn-Yahr stage; (2) Non-motor symptoms: UPDRS I, Mini-Mental State Examination (MMSE), rapid eye movement (REM) sleep behavior disorder questionnaire (RBDQ-HK), Rome III functional constipation, Hamilton depression scale (HAMD), Epworth sleepiness score (ESS), Hyposmia rating scale (HRS); (3) Motor complications: Wearing-off was assessed by the Wearing-off Questionnaire-9 (WOQ-9), dyskinesia was assessed by the Rush Dyskinesia rating Scale and UPDRS IV; and (4) Others: Parkinson's disease questionnaire-39 item version (PDQ-39).

All participants gave written informed consent, which was approved by the Expert Committee of Xiangya Hospital of Central South University in China.

### Statistical Analysis

Statistical analysis of data was performed using SPSS 22.0. Unadjusted and adjusted variables of sex, age of onset and disease duration were compared by univariable regression analysis. A multivariable logistic regression model was adapted to further screen predictive factors for the emergence of dyskinesia. To identify potential predictive factors, odds ratios (OR) and 95% confidence intervals (CI) were calculated. In all analyses, *p* < 0.05 were considered significant. Family history, sex, age of onset, disease duration at assessment, exposure to pesticides and personal preferences (cigarette, tea, coffee, wine), LEDD, BMI, UPDRS I, UPDRS II-ON state, UPDRS III-ON state, Hoehn-Yahr stage-ON state, RBD, ESS, constipation, and HRS were entered into the model. A likelihood ratio (LR) stepwise procedure was used with a 0.02 significance level for entry and 0.05 for elimination.

The Cox proportional hazards statistic was used to pairwise compare the 4 groups stratified by LEDD. To adjust for differences among the 4 groups, Hazard ratios (HRs) and *p-*values were adjusted for sex and age of onset.

Comparison among groups classified by the time from onset of PD to the initiation of levodopa was analyzed by the Mann-Whitney test and presented as median and interquartile range (IQR).

## Results

### Demographic and Clinical Features

The characteristics of our initial sample stratified by the presence and absence of dyskinesia are presented in Table 1. Patients with dyskinesia were characterized by being younger, being female and having a longer disease duration (*p* < 0.001). Therefore, the other variables were assessed on the basis of adjusting for age of onset, sex and disease duration. Our data showed that dyskinesia was negatively related to BMI and positively associated with LEDD (*p* < 0.05). Regarding the severity of disease, dyskinesia was positively related with UPDRS II in ON-state (adjusted OR 1.036; *p* < 0.05) and Hoehn-Yahr stage in both ON-state (adjusted OR 1.562; *p* < 0.001) and OFF-state (adjusted OR 1.359; *p* < 0.05), but negatively related with UPDRS III in ON-state (adjusted OR 0.983; *p* < 0.05), indicating that dyskinesia patients had a better response to levodopa therapy. Sub-analysis of UPDRS III revealed that compared to postural instability subtypes, PD patients with tremor-dominant or intermittent subtypes had less chance to develop dyskinesia (*p* < 0.002). A cross-comparison of motor complications indicated that the incidence of wearing-off and ON-OFF phenomena was positively correlated with dyskinesia (*p* < 0.05). Comparisons of non-motor symptoms showed that RBD, constipation and depression were positively related with dyskinesia (*p* < 0.05). Dyskinesia patients had a poorer quality of daily life (*p* = 0.001). The evaluation of MMSE, ESS, hyposmia, positive family history, exposure to pesticides and personal preferences (cigarette, tea, wine) revealed that there were no significant differences between the two groups after adjustment.

**Table 1:** Cross-sectional analysis of characteristics of PD patients with and without dyskinesia.

**Characteristics**	**Unadjusted OR (95% CI)**	***p*-value**	**Adjusted OR (95% CI)**	***p***	**Multivariate OR (95% CI)**	***p***
Female	1.529 (1.206–1.938)	< 0.001^*^			1.974 (1.363–2.860)	< 0.001^*^
Age at onset	0.946 (0.935–0.958)	< 0.001^*^			0.946 (0.928–0.965)	< 0.001^*^
Disease duration	1.179 (1.149–1.210)	< 0.001^*^			1.104 (1.059–1.152)	< 0.001^*^
^a^BMI	0.908 (0.870–0.947)	< 0.001^*^	0.945 (0.905–0.987)	< 0.011^*^		
^a^LEDD	1.003 (1.002–1.003)	< 0.001^*^	1.003 (1.002–1.003)	< 0.001^*^	1.003 (1.003–1.004)	< 0.001^*^
^a^UPDRS I	1.087 (1.028–1.150)	0.003^*^	1.053 (0.990–1.120)	0.100		
^a^UPDRS II-ON	1.063 (1.038–1.089)	< 0.001^*^	1.036 (1.007–1.066)	0.015^*^		
^a^UPDRS II-OFF	1.055 (1.027–1.085)	< 0.001^*^	1.023 (0.990–1.058)	0.169		
^a^UPDRS III-ON	1.000 (0.989–1.012)	0.966	0.983 (0.970–0.997)	0.014^*^	0.956 (0.939–0.973)	< 0.001^*^
^a^UPDRS III-OFF	1.021 (1.008–1.033)	0.001^*^	1.006 (0.992–1.020)	0.391		
^a^Motor subtype						
PIGD	Reference	–	Reference	–		
TD	0.462 (0.315–0.678)	<0.001^*^	0.529 (0.351–0.796)	0.002^*^		
Indeterminate	0.641 (0.510–0.806)	<0.001^*^	0.653 (0.514–0.830)	<0.001^*^		
^a^Hoehn-Yahr stage-ON	1.903 (1.574–2.301)	<0.001^*^	1.562 (1.250–1.951)	<0.001^*^	2.343 (1.741–3.155)	<0.001^*^
^a^Hoehn-Yahr stage-OFF	1.705 (1.357–2.144)	<0.001^*^	1.359 (1.042–1.773)	0.024^*^		
^a^HAMD	1.063 (1.041–1.085)	<0.001^*^	1.694 (1.310–2.191)	<0.001^*^		
^a^PDQ-39	1.014 (1.009–1.018)	<0.001^*^	1.008 (1.003–1.013)	0.001^*^		
^a^MMSE	1.025 (0.750–1.402)	0.876	0.953 (0.672–1.351)	0.786		
^a^ESS	0.911 (0.709–1.171)	0.466	0.814 (0.617–1.073)	0.145		
^a^RBD	1.620 (1.280–2.051)	<0.001^*^	1.465 (1.126–1.905)	0.004^*^		
^a^Hyposmia	1.216 (0.961–1.538)	0.104	1.204 (0.933–1.554)	0.154		
^a^Constipation	1.440 (1.136–1.825)	0.003^*^	1.330 (1.014–1.745)	0.040^*^		
^a^Wearing-off	4.258 (3.310–5.477)	<0.001^*^	3.444 (2.641–4.493)	<0.001^*^		
^a^ON-OFF	5.833 (4.526–7.519)	<0.001^*^	4.488 (3.433–5.866)	<0.001^*^		
^a^Positive family history	1.080 (0.726–1.605)	0.705	0.767 (0.486–1.209)	0.253		
^a^Cigarette smoking	0.685 (0.503–0.933)	0.016^*^	0.923 (0.634–1.344)	0.677		
^a^Tea consumption	1.212 (0.852–1.725)	0.285	1.349 (0.922–1.973)	0.123		
^a^Wine consumption	0.720 (0.520–0.995)	0.720	0.889 (0.615–1.286)	0.532		
^a^Pesticide exposure	0.680 (0.471–0.982)	0.040^*^	0.857 (0.580–1.267)	0.439		

a *Adjusting for age of onset, sex and disease duration*.

* *There was a significant difference (p < 0.05); w/: with; w/o: without; y: years*.

A multivariable logistic analysis prediction model showed that younger age of onset, high LEDD, being female, high Hoehn-Yahr stage in ON-state and low UPDRS III score in ON-state were risk factors for the prediction of dyskinesia. The correct classification was 85.7% for dyskinesia according to the receiver operating characteristic (ROC) curve.

### Association Between Levodopa Equivalent Daily Dose and Risk of Dyskinesia

**Figure 1:**
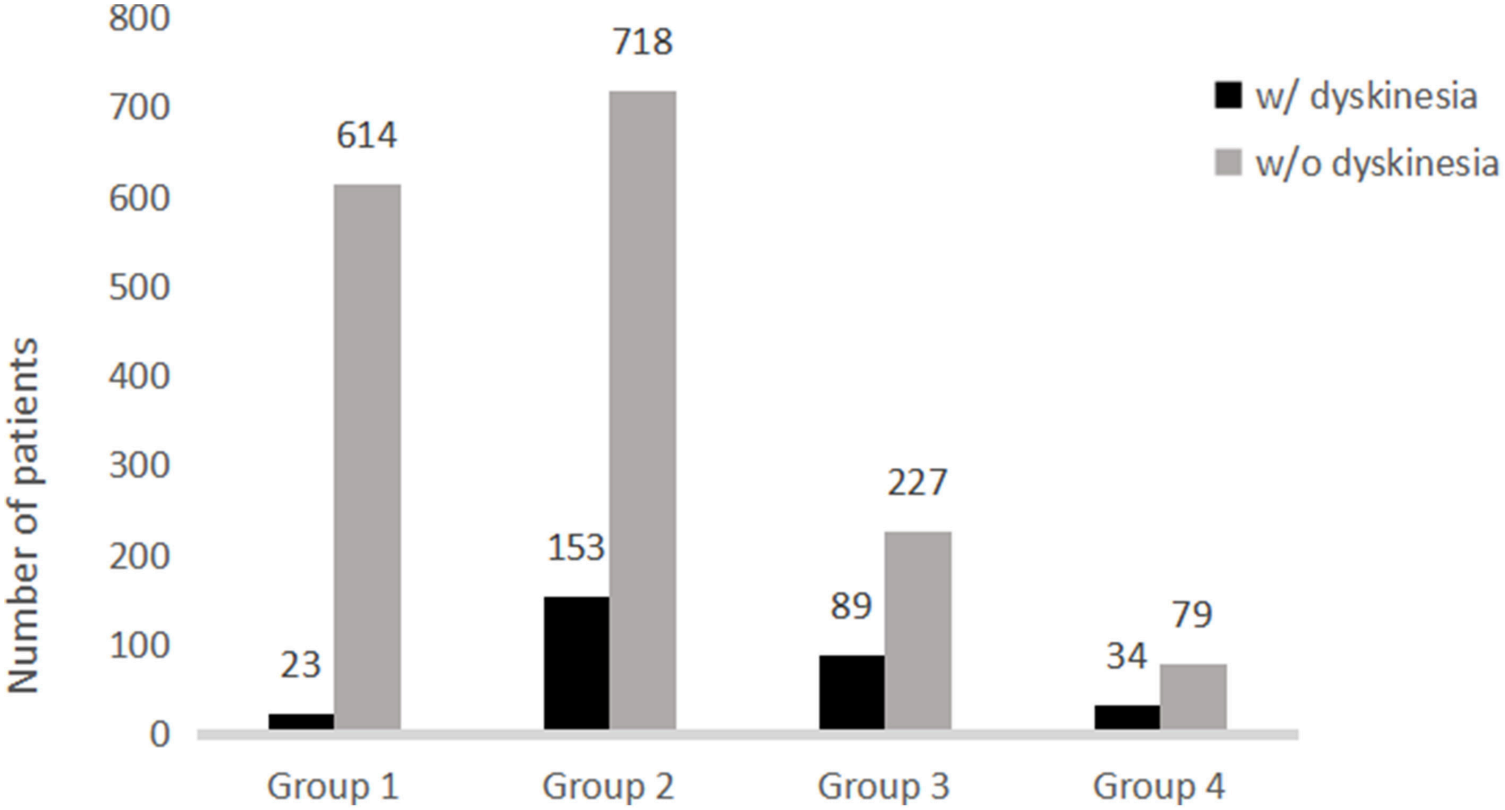
Distribution of patients with and without dyskinesia stratified by levodopa equivalent daily dose.

Further analysis of the correlation between the development of dyskinesia and actual LEDD at the time of dyskinesia or at the time of assessment if the patients did experienced dyskinesia are shown in Figure 1 and Table 2. Among 1,937 patients, the frequency of dyskinesia at assessment was 3.6% (23/637) in Group 1, 17.6% (153/871) in Group 2, 28.2% (89/316) in Group 3, and 30.1% (34/113) in Group 4 (Figure 1). Figure 1 also showed that the LEDD of our patients mainly concentrated in Group 2 (301–600 mg/d). HR indicated that the incidence of dyskinesia increased remarkably when LEDD exceeded 300 mg/d (Table 2, *p* < 0.05).

**Table 2:** Statistical analysis of time to dyskinesia classified by levodopa equivalent daily dose.

**Groups**	**n/n**	**HR^a^**	**95% CI**	**Reduction in LID**	***p*-value**
1 vs. 2	637/871	0.321	0.207–0.498	67.9%	<0.001^*^
1 vs. 3	637/316	0.280	0.176–0.446	72.0%	<0.001^*^
1 vs. 4	637/113	0.245	0.142–0.423	75.5%	<0.001^*^
2 vs. 3	871/316	0.842	0.647–1.095	15.8%	0.200
2 vs. 4	871/113	0.783	0.537–1.140	21.7%	0.202
3 vs. 4	316/113	0.934	0.626–1.395	6.6%	0.740

PD patients (N = 1937) were stratified into 4 groups based on the actual LEDD at the onset of dyskinesia or at the evaluation if the patient had not experienced dyskinesia: Group 1: ≤ 300 mg/day (n = 637); Group 2: 301–600 mg/day (n = 871); Group 3: 601–900 mg/day (n = 316); Group 4: >900 mg/day (n = 113); HR, hazard ratio; CI, confidence interval;

a An HR < 1 indicates a smaller risk for dyskinesia in the group mentioned first;

* *There was a significant difference (p < 0.05)*.

### Correlation Between Initiation of Levodopa and Onset of Dyskinesia

**Figure 2:**
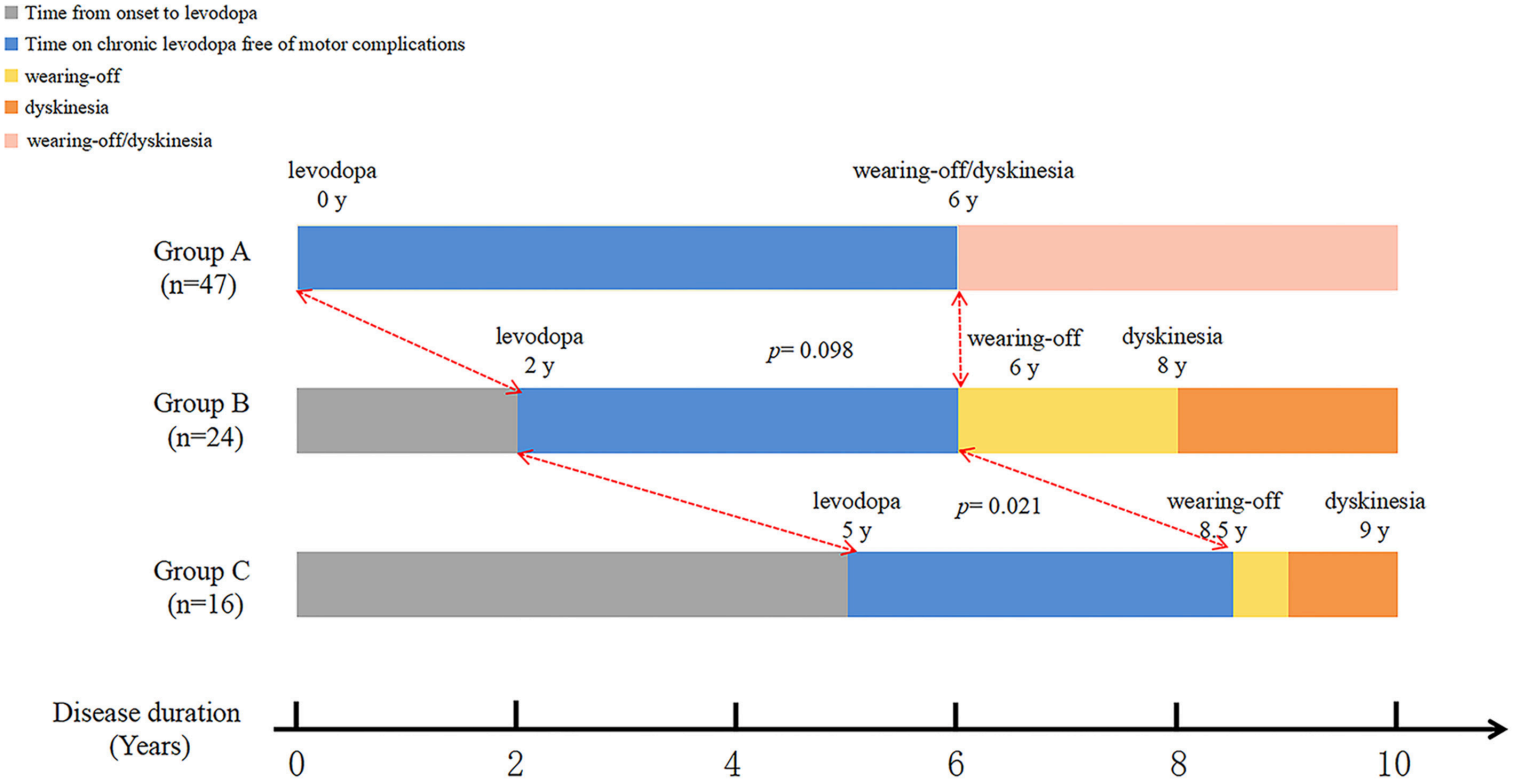
Graphical presentations of the relationship between initiation of levodopa therapy and emergence of dyskinesia.

Longitudinal analysis of patients with dyskinesia (*n* = 87) revealed that the median disease duration at the emergence of wearing-off and dyskinesia were comparable among three groups: time from PD onset to levodopa: 0 years (IQR 0–1) vs. 2 years (IQR 2–2) vs. 5 years (IQR 4–8); time from PD onset to wearing-off: 6 (IQR 5–8) vs. 6 years (IQR 6–9) vs. 8.5 years (IQR 6–10.75); time from PD onset to dyskinesia: 6 (IQR 5–8) vs. 8 years (IQR 5.25–10) vs. 9 years (IQR 7–10) (Figure 2). Interestingly, although the initiation time was different, the emergence of dyskinesia was similar among the three groups (Figure 2 and Table 3, *p* > 0.05). Unlike dyskinesia, if the initiation time of levodopa exceeded 4 years after PD onset, the appearance of wearing-off would delay (Table 3).

**Table 3:** Statistical analyses of the relationship between initiation of levodopa and onset of motor complications.

**Group**	**Time from onset to levodopa initiation (y)**	***p***	**Time from onset to wearing-off (y)**	***p***	**Time from onset to dyskinesia (y)**	***p***
^d^A vs. B	0 vs. 2.0	<0.001	6.0 vs. 6.0	0.370	6.0 vs. 8.0	0.228
^d^B vs. C	2.0 vs. 5.0	<0.001	6.0 vs. 8.5	0.149	6.0 vs. 9.0	0.633
^d^A vs. C	0 vs. 5.0	<0.001	6.0 vs. 8.5	0.015*	8.0 vs. 9.0	0.103

Grouped according to time from PD onset to levodopa: Group A: < 2 years; Group B: 2–3 years; Group C: ≥4 years; d: Calculated by Mann-Whitney tests; *: Results were significant; y, years; vs., versus.

## Discussion

The prevalence of dyskinesia was 25.74% after 5 years of treatment with levodopa and 39.26% after 10 years in our population, which was much higher than Wei Chen's reports from the Mainland Chinese population, which were 12.8 and 19.3%, respectively (5), but still lower compared to previous reports from other countries (1, 2). Participants with higher LEDD or at an advanced stage tended to take levodopa more frequently and take a combination of several medications in the current study, which could have impacted the development of dyskinesia as well (1).

Patients with dyskinesia in this study were characterized by young age of PD onset, being female, long disease duration, low BMI and high LEDD; these findings are consistent with other studies (5, 16). We did not find significant differences in other demographic features, while some studies reported that coffee or nicotine were correlated with dyskinesia (17, 18). Cigarette smoking was relatively common (22.6%) and coffee consumption was rare (3.1%) in our population. Consistently, tremor symptoms were a protective factor against dyskinesia in our patients (19). As several studies have suggested, PD patients with resting tremor as an initial manifestation had a slower progression of the disease, and we speculated that patients with dyskinesia might have a faster progression as well (20), but long-term follow-up is needed to verify this point.

Non-motor symptoms also negatively impact the quality of life of patients. Young et al. showed that dyskinesia was significantly related to RBD in a multivariate analysis (21), which was in line with our dyskinesia patients. Nicola Pavese detected reduced presynaptic nigrostriatal dopaminergic function and increased microglial activation in SN of patients with idiopathic RBD (22), indicating a shared or overlapping pathogenesis for RBD and dyskinesia. Our results also suggested that patients with dyskinesia bear a great psychological burden, and we should concentrate on psychological symptoms in clinical practice. In addition, poor quality of daily life and depression might form a vicious circle. Constipation, which has been linked to gastrointestinal flora imbalance, was more frequent in our dyskinesia patients. Recently, a novel study established that particular microbial communities were required for hallmark motor and gastrointestinal tract dysfunction in a mouse model of PD (23). Although hyposmia is also a common non-motor symptom and usually emerges earlier than motor symptoms, we did not find any significant correlation between dyskinesia and hyposmia. Other studies have also suggested that anosmia was not associated with dyskinesia (24).

Numerous studies have proposed that higher doses of levodopa and longer disease durations could increase the risk of dyskinesia (25). Lee's study supported the interplay of presynaptic dopaminergic denervation and dyskinesia through dopamine transporter (DAT) imaging (26). Without adequate buffering effects from DAT, exogenous levodopa would stimulate dopamine receptors non-physiologically (26). Our patients with dyskinesia were more sensitive to exogenous levodopa. However, PD patients do appear to have altered sensitivity to developing dyskinesia, which may provide clues to other pathophysiological mechanisms. LEDD was a relatively controllable variable by clinicians. Making clear the association between LEDD and dyskinesia would be beneficial to both PD patients and physicians. We found that dyskinesia was levodopa dosage-dependent, in that the incidence increased when the daily LEDD exceeded 300 mg/d. This was different from another study, which reported that 400 mg/d was the warning line (27). The possible explanations might be racial differences, lower BMI of Chinese people, use of LEDD instead of actual levodopa dose, or other environmental and social factors. We also analyzed our data using LEDD = 400 mg/d as a demarcation line, and it was statistically significant (data not shown). To minimize the occurrence of dyskinesia, we recommend no more than 300 mg/d as a safe dosage level, although each patient requires individualized doses of LEDD to manage symptoms and retain an acceptable quality of life.

For years, there had been several debates on whether we should delay the initiation of levodopa until dyskinesia was obvious or the quality of life was reduced substantially (28). Some studies proposed that no significant differences were observed between the occurrence of dyskinesia and levodopa initiation after adjusting for certain variables, such as age and disease duration (29, 30). Gianni Pezzoli and colleagues compared patients from Ghana and Italy and concluded that dyskinesia was not associated with the duration of levodopa therapy but rather with longer disease duration (31); however, racial differences were not taken into consideration in their study, and such problems did not exist in our study. To our knowledge, we were the first to longitudinally demonstrate that the early initiation of the levodopa group was characterized by a long “honeymoon phase” (time on chronic levodopa free of motor fluctuations) in China. Conversely, patients with late initiation of levodopa suffered from motor symptoms of PD without medication for a long time and had a relatively short “honeymoon phase,” which would reduce the overall quality of life. Furthermore, the appearance of dyskinesia was correlated with disease duration rather than time of levodopa initiation in this patient population.

Collectively, as one of the largest studies, we conclude that young age of onset, female sex, long disease duration, high LEDD, high Hoehn-Yahr stage in ON-state and low UPDRS III score in ON-state could serve as predictors for dyskinesia for Mainland Chinese populations. Physicians should be cautious if the LEDD exceeds 300 mg/d. It was better for patients to start levodopa with low doses and multiple administration times. We recommend the initiation of therapy in the early stage instead of delaying the use of levodopa or using the dosage that does not obtain a satisfactory effect.

Several limitations of this study should be noted. First, we did not perform a multivariable analysis in the longitudinal study since the limitation of our sample size, and larger population cohorts will be needed in our future study to further verify the present findings. Second, multiple studies have identified that genetic factors contribute to PD in our populations (32–35), and genetic variations might predispose certain patients to dyskinesia but were not taken into consideration in the current study. Biomarker collection and long-term follow-up would be our next aim. Third, the association between the levodopa response cycle and subtypes and the topographical organization of dyskinesia remain unclear. These factors were not within the scope of our study. Fourth, studies concerning specific types and different frequencies of medication administration should be investigated in the future.

## Ethics Statement

All participants gave written informed consent, which was approved by the Expert Committee of Xiangya Hospital of Central South University in China.

## Author Contributions

XZ and LF were involved in writing the manuscript, organization and execution of both the research project and statistical analysis. JG and BT were involved in the execution and organization of the research project. JG were involved in the review and critique of statistical analysis. XZ, QX, and QS were involved in the patients' follow-up and evaluation. XZ, HP, and JT were involved in figure modification. RY was involved in the review and critique of statistical analysis. XY was involved in outpatient management. All authors reviewed the manuscript.

### Conflict of Interest Statement

The authors declare that the research was conducted in the absence of any commercial or financial relationships that could be construed as a potential conflict of interest.
